# Effects of *APOE* isoforms in diabetic nephropathy patients of South India

**DOI:** 10.1007/s00592-024-02374-2

**Published:** 2024-10-17

**Authors:** Preethi Basavaraju, Puthamohan Vinayaga Moorthi, Arun Meyyazhagan, Ilakkiyapavai Devaraj, Kavipriya Babu, Emanuele Panza, Antonio Orlacchio

**Affiliations:** 1 Biomaterial and Nano-materials Laboratory, Department of Human Genetics and Molecular Biology, Bharathiar University, Coimbatore, Tamil Nadu India; 2 Dipartimento di Medicina e Chirurgia, Università di Perugia, Piazza L. Severi - Edificio B, Piano 1, Sant’Andrea delle Fratte, Perugia 06132 Italy; 3 Dipartimento di Scienze Mediche e Chirurgiche, Università di Bologna, Bologna, Italy; 4 Laboratorio di Neurogenetica, Centro Europeo di Ricerca sul Cervello (CERC), Istituto di Ricovero e cura a Carattere Scientifico (IRCCS) Fondazione Santa Lucia, Rome, Italy

**Keywords:** Diabetic nephropathy, Apolipoprotein-E, Allelic variants, Proteinuria and lipid profile

## Abstract

**Background:**

Diabetic nephropathy (DN) is a grave complication and the most common renal dysfunction of diabetes mellitus. Genetic factors, including Apolipoprotein E (*APOE*) isoforms, have been implicated in the pathogenesis of DN.

**Methods:**

A total of 577 type 2 Diabetes mellitus subjects were categorized into diabetes non-nephropathic (Controls: *n* = 321), diabetes nephropathic (DN: *n* = 256) groups. Demographic, clinical, and biochemical parameters including age, BMI, lipid profiles (TC, LDL-C, HDL-C, TG), glucose metabolism (plasma glucose, HbA1c, serum insulin), renal function (UACR, PCR), and blood pressure (SBP, DBP) were assessed. *APOE* variant frequencies were determined using restriction fragment length polymorphism (RFLP) analysis, validated against Hardy-Weinberg equilibrium (HWE), and statistically correlated with each clinical and biochemical parameter.

**Results:**

The DN group had an increased prevalence of hypertension, fatty liver, and dyslipidemia compared to the Control group. Biochemical analyses revealed elevated levels of TC (213.41 mg/dL vs. 189.32 mg/dL), LDL-C (134.46 mg/dL vs. 107.56 mg/dL), and reduced HDL-C (58.13 mg/dL vs. 65.32 mg/dL) in DN cases compared to Controls (all *p* < 0.0001). The *APOE* variants distribution showed a significant increase in E2 allele frequency (69.1% vs. 15.3%) and corresponding homozygous genotype (E2/2: 42.2% vs. 5.6%) in DN cohorts.

**Conclusion:**

The study found a higher frequency of E2 allele in the DN group compared to Controls, though no statistically significant risk of DN was linked to this allele. The results suggest a potential association for *APOE* polymorphisms, requiring broader studies to clarify the role of *APOE* polymorphisms in DN susceptibility.

**Supplementary Information:**

The online version contains supplementary material available at 10.1007/s00592-024-02374-2.

## Introduction

Diabetic nephropathy (DN) is a significant global health issue, with prevalence rates in India ranging from 0.9 to 62.3% [1]. Although these estimates are sporadic, DN affects almost one-third of the diabetic population, influenced by vasoactive renal factors and metabolic abnormalities [2]. At clinical levels DN is characterized by specific changes in kidney structure and function, including progressive decline in renal functions and persistent albuminuria [3–5]. Notably, albuminuria ranging from microalbuminuria (~ 30–300 mg/day) to nephrotic-range proteinuria (> 3.5 g/24 hours) mark the severity of the disease progression [6]. According to the 2021 International Diabetes Federation (IDF) Atlas, approximately 783 million people globally are affected by diabetes [7], with 20–30% of these cases progressing to microalbuminuria, and fewer than half developing into overt nephropathy [8]. The American Diabetes Association (ADA) and the Kidney Disease: Improving Global Outcomes (KDIGO) recommend routine evaluation of renal function and albuminuria in diabetic patients at diagnosis [9], followed by annual assessments using the albumin-to-creatinine ratio (ACR) and serum-creatinine-based estimated glomerular filtration rate (eGFR) [10, 11].

Genetic predisposition is believed to play a significant role in the prognosis of DN, although the precise molecular mechanisms remain unclear. Among the genetic factors, the influence of Apolipoprotein E (*APOE*) polymorphisms on DN development has been extensively studied in both type 1 diabetes mellitus (T1DM) and type 2 diabetes mellitus (T2DM) populations [12–14]. The *APOE* gene is polymorphic at the 112th (rs429358) and 158th (rs7412) amino acid positions [15, 16], resulting in three allelic variants - E2, E3, and E4 within the *APOE* locus that give rise to six genotypes - E2/2, E2/3, E2/4, E3/3, E3/4, and E4/4 [17]. The worldwide frequencies of the *APOE* alleles are E2 = 8.4%, E3 = 77.9%, and E4 = 13.7%, respectively [18]. *APOE* isoforms exhibit distinct functional traits, where the E3 allele functions normally, while the E2 and E4 alleles show differential affinities for remnant and receptor molecules. This results in reduced total cholesterol and low-density lipoprotein cholesterol (LDL-C) levels in E2 carriers and elevated levels in E4 carriers [19]. Beyond lipid regulation, *APOE* isoforms facilitate cholesterol efflux from foam cells and modulate inflammatory responses and antioxidant activities in blood vessels [20].

*APOE* polymorphisms have been implicated in various clinical conditions, including renal diseases, hyperlipidemia, cardiovascular disease (CVD), coronary heart disease, atherosclerosis, and neurodegenerative diseases [21–23]. Some research outcomes indicate that carriers of the E2 allele with long-standing T1DM experience a significantly higher risk of DN, while other studies did not find a clear association between *APOE* polymorphism and DN in individuals with T1DM. A meaningful study on T1DM showed a 3.1-fold increased risk of DN in E2 carriers [24], whereas European cohort studies found no significant role for *APOE* polymorphisms in DN predisposition [25]. Similar contradictions are also reported with type 2 diabetes mellitus (T2DM) cases, with some studies suggesting a strong association between the E2 allele and DN risk, while others propose a protective role for the E4 allele [26–28]. These discrepancies may arise from differences in DN diagnostic criteria, ethnic diversity, dietary variations, and other genetic or environmental factors. Recent studies suggest a potential role of *APOE* polymorphisms in DN prognosis [29, 30]. However, further research on well characterized and defined DN cohorts is crucial to elucidate this relationship. This study aims to investigate the impact of *APOE* isoforms exclusively on DN cases from South India for a period of four years, potentially providing significant insights into the role of *APOE* polymorphisms, particularly the E2 allele, in the development of DN.

## Methods

### Study design and subject recruitment

A total of 798 T2DM patients aged ≥ 50 years were initially recruited for this retrospective cohort study, conducted over a four-year period (2018–2022) at the Department of Human Genetics and Molecular Biology, Bharathiar University, Tamil Nadu, India, in collaboration with several diabetes care centres in South India. Participants were recruited from September 2018 to December 2022, ensuring a diverse and representative sample. Demographic parameters such as age, sex distribution, body mass index (BMI), and waist-to-hip ratio were recorded, along with clinical attributes including diabetes duration, presence of hypertension, fatty liver, dyslipidemia, and cardiovascular disease (CVD)-related complications. The subjects were then categorized into two major groups: the DN group and the Control group.

The DN group included 256 patients diagnosed with DN based on clinical characteristics, including persistent albuminuria (urinary albumin-to-creatinine ratio (UACR) ≥ 30 mg/g), declining estimated glomerular filtration rate (eGFR), and other relevant clinical symptoms evident during physical examination. Additional confirmation methods for DN included renal ultrasound, computed tomography (CT), magnetic resonance imaging (MRI) scans, and biopsy examinations when necessary. Inclusion criteria for the DN group required participants to have a diagnosis of T2DM for at least five years. The Control group included 321 diabetic patients with no evidence of DN, characterized by the absence of persistent albuminuria (UACR < 30 mg/g) and normal eGFR. Inclusion criteria for the Control group required participants to have T2DM for at least five years, be aged 50 years or older, and maintain normal eGFR. Exclusion criteria for both groups included any history of sexually transmitted diseases (STDs) severe viral infections or malignancies.

### Sample collection and ethical considerations

3.5 ml of peripheral blood and 2 ml of urine samples was collected from all 577 participants both in the fasting state and postprandially. The samples were collected to perform biochemical assessments and *APOE* genotyping. Samples were processed immediately for biochemical analysis, with remaining samples stored at -80 °C for subsequent genetic analysis. Ethical approval for the study was granted by the Institutional Human Ethics Committee of Life Sciences at Bharathiar University (Approval Number: BUHEC-009/2018), and informed consent was obtained from all participants. The study adhered to ethical protocols outlined in the Declaration of Helsinki 1964 and guidelines from the World Medical Association (WMA, 2000) [31], ensuring participant confidentiality and diligent informed consent procedures.

### Biochemical assessments

Biochemical profiles were assessed and recorded for each participant. The measurements included total cholesterol (TC), low-density lipoprotein cholesterol (LDL-C), high-density lipoprotein cholesterol (HDL-C), triglycerides (TG), plasma glucose, glycated haemoglobin (HbA1c), serum insulin, urine albumin-to-creatinine ratio (UACR), protein-to-creatinine ratio (PCR), systolic blood pressure (SBP), and diastolic blood pressure (DBP). Lipid profiles were determined using enzymatic colorimetric assays on a clinical chemistry analyser (Cobas c 111 analyser – Roche Diagnostics, India). Plasma glucose was measured with the glucose oxidase method using a Accu-check active blood glucose meter (Accu-Check, India). HbA1c levels were assessed by high-performance liquid chromatography (HPLC) on a D-10 Haemoglobin Testing System (Bio-Rad Laboratories India). Serum insulin was quantified via electrochemiluminescence immunoassay (ECLIA) (Cobas c 111 analyser – Roche Diagnostics, India). UACR and PCR were evaluated using urine samples with immunoturbidimetric and Jaffe methods, respectively, on AU480 Chemistry Analyzer (Beckman Coulter, USA). Blood pressure was measured using an automated oscillometric HEM 7120 - blood pressure monitor (OMRON Healthcare India).

### Molecular analysis

#### DNA extraction

40–60 µg of genomic DNA was extracted using the modified Miller et al. (1988) salting-out method [32] from the 1 ml of whole blood samples collected from each of the study subjects. In the final step, DNA pellets were extracted out by treating the supernatants with an equal volume of ice-cold absolute alcohol. The pellets were then prewashed with 85% ethanol, air-dried for 10–15 min, and redissolved in ~ 150 µl Tris EDTA buffer immediately for better yield.

#### *APOE* genotype

The genomic DNA extracted was used as a template to amplify the 243-bp-long *APOE* gene fragment on a thermal cycler (Eppendorf Mastercycler Nexus Gradient Thermal Cycler, Eppendorf SE, Germany). The reaction cycle was set to begin with a denaturation step at 94^o^C for 5 minutes, followed by 30 cycles of primer annealing at 60.5^o^C for 1 minute, extension at 72^o^C for 2 minutes, and denaturation at 94^o^C for 1 minute [33]. The reaction mix contained 2.5µl of the template, 12.5µl 2X ready-to-use Master Mix (ReadyMix Taq PCR Reaction Mix with MgCl2, Sigma Aldrich or Merck, India), 1.5 pmol/ml forward (3’-GTTTTGAGCATTTCAT-5’) and reverse (3’-GAACGGGGGTCGAACT-5’) oligonucleotide sequences (Bioserve Biotechnologies, Pvt. Ltd., India), and was made up to a final volume of 25 µl with nuclease-free water. The 300ng amplified PCR products were sequentially restriction digested using 3U *Hha-I* restriction enzyme (New England Biolabs Inc. US) at 37˚C for 12–16 h to yield varied lengths of DNA fragments visualized on a 3% agarose gel (Supplementary Fig. 1A). These polymorphic fragments were used to interpret the *APOE* genotypes in each study subject.

### Statistical analysis

Categorical variables such as sex, presence of hypertension, fatty liver, dyslipidemia, and CVD-related complications were compared between groups using chi-square tests (IBM SPSS Statistics 28.0.1), while continuous variables such as age, BMI, and biochemical profiles were analysed using student’s t-test or Mann Whitney U-test (IBM SPSS Statistics 28.0.1) and *p*-value ≤ 0.05 indicated the statistical significance. The *APOE* variant distributions obtained from Chi-square (GENALEX) were compared between DN and Control subjects using z-test at *p* ≤ 0.05; 95% CI, with *p*-values < 0.05 indicating significant deviations from Hardy-Weinberg equilibrium (HWE). Logistic regression analysis from the statsmodels library (Python) was used to quantify the relative likelihood of DN development in each risk *APOE* allele with E3 as the reference group. The OR and 95%CI were adjusted for the covariates (age, sex, levels of TC, LDL-C, HDL-C, TG, Glucose, HbAIC and Serum Insulin) and statistical significance was set at *p* < 0.05. Additionally, Receiver Operating Characteristic (ROC) curve analysis (scikit-learn, Python) assessed the predictive ability of *APOE* genotypes for DN by leveraging tools like scikit-learn. The Area under the Curve (AUC) (> 0.7) derived from the ROC curve served as an indicator of the accuracy of the predictive model. The variations in biochemical profiles between the genotypes were assessed using One-way analysis of variance (ANOVA) followed by Tukey’s post-hoc test (GraphPad PRISM 10.3.1). The data was validated using Pearson’s correlation Heatmap (Python) to study the associations between each clinical, biochemical and genetic parameter observed in DN cohorts.

## Results

### Clinical characteristics

The demographic and clinical characteristics of all subjects are listed in Table 1. There were no significant differences observed between Control and DN groups relating to the age, BMI and the waist hip ratios (*p*-values: 0.397, 0.230, 0.520, respectively). There is a significant difference in sex distribution between the Control and DN groups. The DN group has a higher proportion of females compared to the Control group, with *p*-values indicating significant differences for males (*p* = 0.0061) and females (*p* = 0.0118). Hypertension was markedly more prevalent in the DN group, with 98 cases compared to just 33 cases in the Control group (*p* < 0.0001). Fatty liver was observed exclusively in the DN group, affecting 26 individuals (*p* < 0.0001), while dyslipidemia was significantly higher in DN subjects with 78 cases as compared to only 3 cases in Control group (*p* < 0.0001). Microalbuminuria and macroalbuminuria were present only in the DN group (*p* < 0.0001 for both). Despite these differences, there was no significant variation observed for the rates of obesity and the incidence of cardiovascular disease-related complications between the Control and DN groups (*p*-values: 0.763, 0.499, respectively). The cumulative outcomes of biochemical profile reveal several significant differences between the Control and DN groups. DN patients exhibit markedly higher levels of TC at 213.41 ± 31.3 mg/dL compared to 189.32 ± 12.33 mg/dL in Controls (*p* < 0.0001), along with elevated LDL-C at 134.46 ± 24.78 mg/dL versus 107.56 ± 11.20 mg/dL in Controls (*p* < 0.0001). Conversely, HDL-C is significantly lower in DN patients (58.13 ± 12.19 mg/dL compared to 65.32 ± 17.80 mg/dL, *p* < 0.0001). Additionally, TG levels are higher in DN patients (158.49 ± 33.24 mg/dL vs. 145.59 ± 12.87 mg/dL, *p* < 0.0001). Plasma glucose levels are elevated in the DN group (147.42 ± 48.72 mg/dL vs. 135.17 ± 30.46 mg/dL, *p* = 0.0002), and HbA1c levels are significantly higher (49.14 ± 18.17 mmol/mol vs. 45.48 ± 13.33 mmol/mol, *p* = 0.0055). DN patients also show higher serum insulin levels (27.81 ± 6.98 µU/mL vs. 25.34 ± 8.59 µU/mL, *p* = 0.0002), reflecting increased insulin resistance. Furthermore, both UACR and PCR are significantly elevated in DN patients (43.95 ± 3.98 mg/g vs. 22.25 ± 6.50 mg/g, *p* < 0.0001 and 27.19 ± 9.18 mg/mmol vs. 12.88 ± 8.99 mg/mmol, *p* < 0.0001, respectively). Systolic blood pressure is slightly higher in the DN group (127.43 ± 10.59 mmHg vs. 124.98 ± 9.81 mmHg, *p* = 0.0042) and diastolic blood pressure is also notably elevated (92.17 ± 6.53 mmHg vs. 88.57 ± 2.76 mmHg, *p* < 0.0001). These findings collectively indicate that DN is associated with significant dyslipidemia, impaired glycemic control, increased insulin resistance, and elevated markers of renal impairment.

**Table 1:** Baseline demographic and clinical characteristics of all study subjects

Characteristics		Controls	DN	*p*-value
N^b^		321	256	-
**Demographics** ^**a, b**^				
Age (years)		69.94 ± 10.35	70.66 ± 9.84	0.397
BMI		31.56 ± 1.2	31.37 ± 2.15	0.230
WHR (cm)		0.80 ± 1.22	0.89 ± 2.12	0.520
Sex	M	169	95	0.0061**, 0.0058**
	F	152	161	0.0118**, 0.0122**
**Clinical Characteristics** ^**b**^				
Hypertension		33	98	< 0.0001**
Fatty liver		0	26	< 0.0001**
Obesity		35	47	0.0183**, 0.0194**
Dyslipidemia		31	78	< 0.0001**
Microalbuminuria		0	184	< 0.0001**
Macroalbuminuria		0	72	< 0.0001**
CVD related complications		56	79	0.0009**, 0.0010**
**Biochemical Profile** ^**a**^				
TC (mg/dL)		189.32 ± 12.33	213.41 ± 31.3	< 0.0001*
LDL-C (mg/dL)		107.56 ± 11.20	134.46 ± 24.78	< 0.0001*
HDL-C (mg/dL)		65.32 ± 17.80	58.13 ± 12.19	< 0.0001*
Plasma TG (mg/dL)		145.59 ± 12.87	158.49 ± 33.24	< 0.0001*
Plasma Glucose (mg/dL)		135.17 ± 30.46	147.42 ± 48.72	0.0002*
HbA1c (mmol/mol)		45.48 ± 13.33	49.14 ± 18.17	0.0055*
Serum Insulin (µU/mL)		25.34 ± 8.59	27.81 ± 6.98	0.0002*
UACR (mg/g)		22.25 ± 6.50	43.95 ± 3.98	< 0.0001*
PCR (mg/mmol)		12.88 ± 8.99	27.19 ± 9.18	< 0.0001*
SBP (mmHg)		124.98 ± 9.81	127.43 ± 10.59	0.0042*
DBP (mmHg)		88.57 ± 2.76	92.17 ± 6.53	< 0.0001*
***APOE *** **Allele Frequency** ^**c**^				
E2		15.3	69.1	< 0.0001^+^
E3		84.4	30.5	< 0.0001^+^
E4		0.3	0.4	0.705
***APOE *** **Genotype Frequency** ^**c**^				
E2/2		5.6	42.2	< 0.0001^+^
E2/3		9.7	27	0.0001^+^
E3/3		81.6	26.2	< 0.0001^+^
E2/4		2.8	4.3	0.167
E3/4		0.3	0.4	0.286
E4/4		0	0	-

- All values are expresses in terms of ^a^Mean±SD, ^b^numbers or ^c^percentage observed

Note:

* Statistical significance was calculated using simple t-test at *p* ≤ 0.05; 95% CI for comparison between the means of Control and DN groups.

^+^ Statistical significance was calculated using z-test at *p* ≤ 0.05; 95% CI by comparing the percentage difference of allele frequencies obtained from Chi-square test for Control and DN groups.

**Statistical significance was assessed by calculating the incidence rate ratio (R1/R2) along with its Poison 95% confidence interval (CI) and associated *p*-value. A *p*-value of less than 0.05 indicates that the ratio R1/R2 is significantly different from 1, suggesting a difference between the rates.

*APOE*: Apolipoprotein E; BMI: Body Mass Index; C: Controls; CVD: Cardiovascular disease; DN: Diabetic Nephropathy; F: Female; HDL-C: High Density Lipoprotein Cholesterol; HbA1c: glycated haemoglobin; LDL-C: Low Density Lipoprotein Cholesterol; M: Male; PCR: Protein-to-creatinine ratio; TC: Total Cholesterol; TG: Triglycerides; UCAR: Urine albumin-to‐creatinine ratio; WHR: Waist-to-hip ratio

### *APOE* - allelic and genotypes frequency

The results from Table 2 show that the HWE was used to validate the allele frequency distributions, and the statistical significance was calculated using z-tests at a *p*-value threshold of ≤ 0.05, and 95% confidence intervals were computed to assess the reliability of the differences in allele frequencies between the groups. There exists a difference in allele frequencies between the sexes within the two groups, with varying distributions of *APOE* genotypes. In both male and female subjects, the frequency of E2/2 genotype is higher in the DN group as compared to the Control group, while the E3/3 genotype is less frequent in the DN group. In the Control group, the most frequent genotype was E3/3, making up 81.6% of the population, followed by E2/3 (9.7%) and E2/2 (5.6%). The E4 allele was rare, with no homozygous E4 genotype observed. Whereas the E2/2 genotype was significantly more common in the DN group as compared to the Control group (42.2% vs. 5.6%, *p* < 0.0001). The E3/3 genotype was less frequent in the DN group (26.2% vs. 81.6%, *p* < 0.0001). Other genotypes show less pronounced differences. These genotype distributions contribute to a significantly more frequent E2 allele in the DN group as compared to the Control group (69.1% vs. 15.3%, *p* < 0.0001), suggesting a significant deviation from HWE. Conversely, the E3 allele being less common in the DN group (30.5% vs. 84.4%, *p* < 0.0001). The E4 allele remains rare in both groups (0.4% in DN vs. 0.3% in Control, *p* = 0.705) (Table 2), suggesting that the E4 allele in the population is in HWE and may not play prominent roles in the genetic susceptibility of DN in this population. These findings suggest that *APOE* polymorphism distribution, particularly the significance of E2 alleles, may play a role in the susceptibility of DN in this population.

**Table 2:** Genotype and allele frequency distributions seen amongst the DN and Control subjects

Groups			*APOE * Genotype							*APOE* Allele Frequency (HWE)			
			2/2	2/3	3/3	2/4	3/4	4/4			2	3	4
**Controls**	*N* − 321	M	4.0	5.3	36.8	0.9	0	0			9.3	36.8	0.9
		F	1.5	4.3	44.9	1.9	0.3	0			5.9	45.2	1.9
**E3 > E2 > E4**													
										χ^2^	0.153	0.844	0.003
**DN**	N 256	M	21.9	18	13.7	2.3	0.4	0			39.8	14.0	2.3
		F	20.3	9	12.5	2	0	0			29.3	12.5	2.0
**E2 > E3 > E4**													
										χ^2^	0.691	0.305	0.004
									*p*-value		< 0.0001*	< 0.0001*	0.705
									95% CI		(0.4947, 0.5813)	(-0.5823, -0.4957)	(-0.0042, 0.0062)

Data are presented in terms of percentage

Note: *The HWE of the allelic variants was analyzed using Chi-square (χ^2^) test, and the statistical significance was calculated using z-test at *p* ≤ 0.05; 95% CI by comparing the percentage difference of allele frequencies obtained from Chi-square test for Control and DN groups

DN: Diabetic nephropathy; M: Male; F: Female; HWE: Hardy–Weinberg equilibrium

### *APOE* Polymorphism and DN risk

**Fig. 1:**
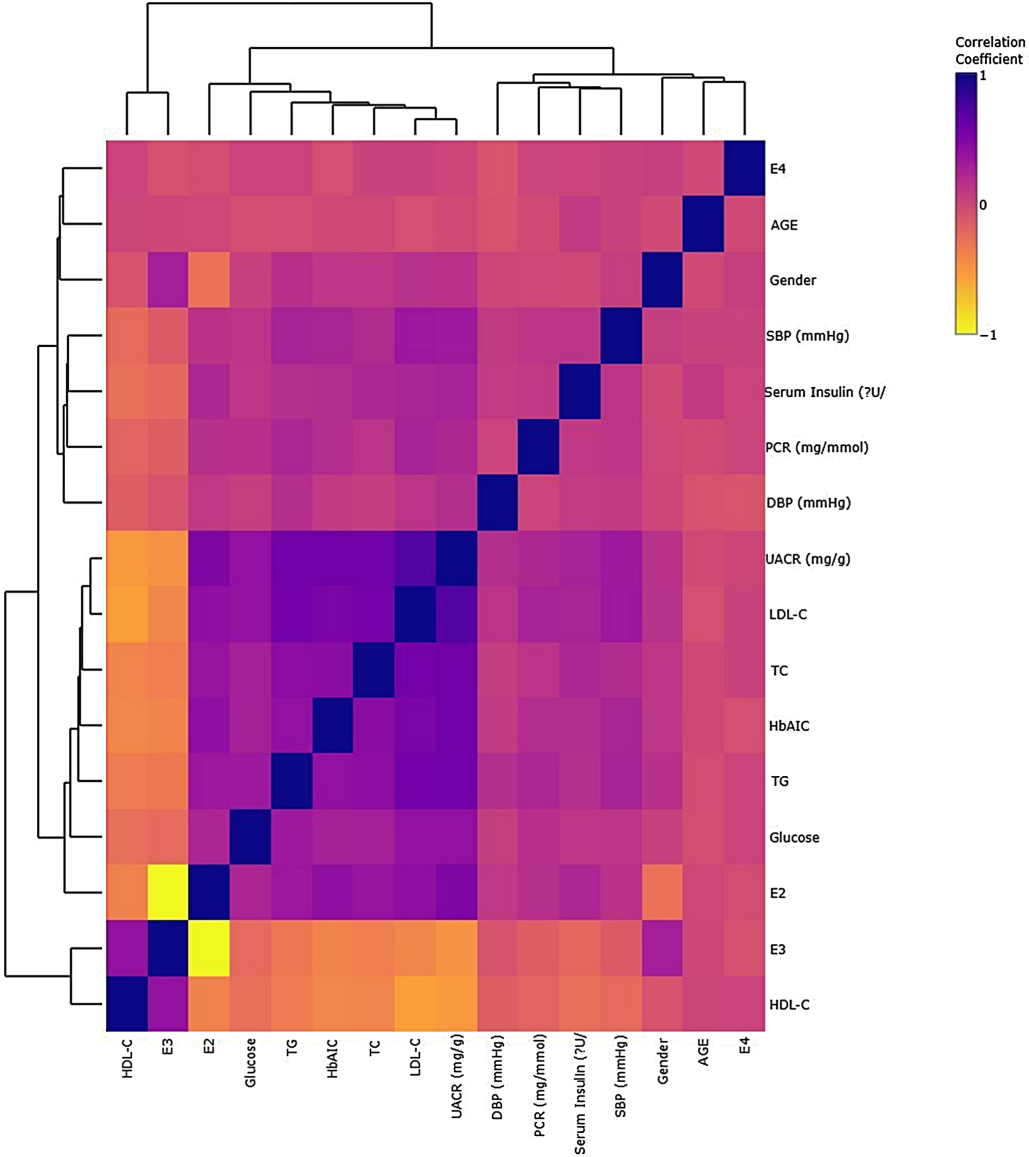
Pearson’s correlation heatmap studied across various parameters in the DN cohorts. The colour gradient form yellow to purple marks the correlation seen from − 1 (negative) to + 1 (positive)

The logistic regression analysis results in Table 3 revealed no statistically significant difference in the risk of DN when comparing E2 to E3 (OR = 3.57, 95% CI: 0.87–14.56, *p* = 1.00) or E4 to E3 (OR = 0.99, 95% CI: 0.45–2.18, *p* = 1.00). The study found that individuals with the E2 allele have a 3.57 times higher risk of developing DN compared to those with the E3 allele. However, the confidence interval does not show a statistically significant difference. Similarly, the study did not find any significant difference in DN risk between the E4 and E3 alleles either, with an odds ratio of 0.99 and a *p*-value of 1.000. The E4 allele does not show a significant difference in DN risk compared to E3. The AUC of 0.7 indicates moderate performance in distinguishing between DN and non-DN cases, suggesting that the predictive accuracy of the model is not very high. Table 4 presents the biochemical and clinical parameters across different *APOE* genotypes in both Control and DN groups. The outcomes highlight distinctive metabolic changes linked to specific *APOE* genotypes in DN development, indicating varying impacts on lipid profiles, glucose metabolism, and insulin sensitivity. Indeed, the DN subjects have markedly altered biochemical and clinical profiles compared to Controls, with significant differences observed in lipid profiles, glucose metabolism, and renal function markers. The E2 genotype is associated with elevated levels of TC, LDL-C, TGs, and plasma glucose, as well as higher insulin levels and renal impairment markers (UACR and PCR) in DN patients. The E3 genotype shows intermediate effects, while E4 exhibits less pronounced changes. These findings suggest that the E2 allele may be linked to more severe metabolic and renal abnormalities in DN, while E3 and E4 genotypes exhibit varying impacts on these parameters. The Fig. 1, illustrating Pearson’s correlation heatmap, reveals that the E2 allele exhibits strong positive correlations with elevated levels of UACR, TG, LDL-C, TC, HbA1C, and plasma glucose in DN patients. Conversely, the E3 allele demonstrates strong negative correlations with these variables in DN cohorts. The observed inverse correlations between TC, TG, LDL-C, and HDL-C align with established lipid relationships. Additionally, a moderate positive correlation between age and the E4 variant is also evident. These findings demonstrate the influence of specific *APOE* genotypes, particularly E2 and E3, on lipid profiles and their contrasting impacts on DN, thereby highlighting the genetic complexities underlying DN pathophysiology.

**Table 3:** Logistic regression analysis comparing odds ratios (OR) and 95% confidence intervals (CI) for different comparisons (E2 vs. E3 and E4 vs. E3) along with the corresponding *p*-values and area under the curve (AUC) from ROC curve analysis

Comparison	*p*-value	OR (95% CI)	AUC
E2 vs. E3	1.000	3.57^a^ (0.87–14.56)	0.7
E4 vs. E3	1.000	0.99^b^ (0.45–2.18)	0.7

Note: The relative odds of the outcome increase (OR more than 1) ^a^, or decrease (OR less than 1) ^b^, observed when the value of the independent variable is increased by one unit

*p-value was calculated using logistic regression model at *p* = 0.05 and 95% Confidence Interval (CI)

AUC: Area Under the curve; CI: Confidence Interval; OR: Odds Ratio; ROC: Receiver Operating Curve

**Table 4:** Variations seen in the biochemical profile evaluated for the different study groups, segregated based on the genotype outcomes

	Controls (*n* = 321)			DN (*n* = 256)			Controls + DN (577)		
	E2	E3	E4	E2	E3	E4	E2	E3	E4
TC	192.40 ± 12.58	189.39 ± 13.73	197.92 ± 0	217.32 ± 19.45**	218.15 ± 19.03***	223.82 ± 0***	211.91 ± 20.87	195.82 ± 19.24	210.87 ± 18.31
LDL-C	105.10 ± 12.29	107.50 ± 10.06	103.70 ± 0	141.71 ± 13.90***	143.16 ± 12.88***	163.95 ± 0***	133.77 ± 20.30	115.47 ± 18.35	133.83 ± 42.60
HDL-C	61.97 ± 20.17	64.68 ± 18.42	56.08 ± 0	59.09 ± 7.84***	59.08 ± 6.37***	55.97 ± 0*	54.05 ± 14.97	58.96 ± 19.65	56.03 ± 0.08
TG	142.83 ± 13.65	146.46 ± 12.59	155.00 ± 0	167.33 ± 10.69***	166.59 ± 10.00***	163.39 ± 0***	162.02 ± 15.21	150.96 ± 14.69	159.20 ± 5.93
Glucose	85.03 ± 33.42	85.98 ± 31.07	107.99 ± 0	188.85 ± 25.84***	189.45 ± 25.73***	217.18 ± 0***	166.34 ± 50.98	109.10 ± 52.53	162.59 ± 77.21
HbAIC	49.10 ± 3.18	48.83 ± 3.19	40.33 ± 0	48.17 ± 15.46***	45.27 ± 16.66***	38.81 ± 0***	44.04 ± 15.84	32.51 ± 10.79	25.57 ± 6.73
Serum Insulin	27.59 ± 12.57	23.36 ± 12.00	22.29 ± 0	30.89 ± 11.16**	29.04 ± 11.28***	52.05 ± 0***	28.00 ± 12.70	16.86 ± 13.51	32.17 ± 28.11
UACR	22.28 ± 5.98	21.74 ± 6.28	25.67 ± 0	57.99 ± 7.83***	57.47 ± 8.82***	66.05 ± 0***	52.41 ± 12.97	37.49 ± 12.77	45.86 ± 28.55
PCR	13.09 ± 9.06	11.78 ± 9.62	14.01 ± 0	39.26 ± 10.34***	39.57 ± 9.99***	36.78 ± 0***	34.45 ± 13.60	22.65 ± 13.28	30.40 ± 9.03
SBP	125.16 ± 10.94	118.26 ± 10.34	126.53 ± 0	126.59 ± 12.06**	128.37 ± 11.72***	127.18 ± 0***	124.11 ± 12.71	110.52 ± 11.45	126.86 ± 14.60
DBP	88.44 ± 13.11	72.00 ± 14.26	84.83 ± 0	97.29 ± 12.22**	86.05 ± 13.79**	89.19 ± 0*	85.37 ± 12.91	72.91 ± 14.23	72.01 ± 1.67

Note: All data are represented in terms of Mean ± SD and was analysed using one-way ANOVA (at *p* < 0.05, 95%CI) and Tukey’s post-hoc test

*Significant difference either compared to the genotype of Control group or Control and (+) DN group for DN group with similar genotype (at *p < 0.05, **p < 0.01, ***p < 0.001)

E2, E3 and E4: Apolipoprotein E isoforms; C: Control; DN: Diabetic Nephropathy; HDL-C: High Density Lipoprotein Cholesterol; LDL-C: Low Density Lipoprotein Cholesterol; PCR: Protein-to-creatinine ratio; TC: Total Cholesterol; TG: Triglycerides; UCAR: Urine albumin-to‐creatinine ratio

## Discussion

The current retrospective cohort study involving 577 participants highlights the impact of *APOE* polymorphisms on DN within the studied population. This study comprehensively compared demographic, clinical, and biochemical characteristics between Control and DN groups. Significant findings include higher prevalence of hypertension, fatty liver, and dyslipidemia in DN subjects. Biochemical analyses revealed elevated levels of TC, LDL-C, TG, plasma glucose, HbA1c, and serum insulin compared to Controls. They also exhibited significantly lower levels of HDL-C. The biochemical and clinical outcomes are consistent with the known characteristics of DN [34]. Renal biomarkers like UACR and PCR were elevated in DN patients, reflecting impaired renal function. These observations are consistent with recent research highlighting atypical forms of DN, often characterized by initial proteinuria, hypertension association, and progressive deterioration over time [35]. In the Control group, the E3/3 genotype was predominant, followed by E2/3 and E2/2. Notably, no E4/4 genotype was observed in both Control and DN cohorts. In contrast, the DN group exhibited a different pattern with a substantial prevalence of the E2/2 genotype, a decrease in E3/3, and a notable presence of E2/3. The allele frequencies demonstrated a significant increase in the E2 allele among DN patients when compared to Controls, while the E3 allele frequency decreased in DN as compared to Controls. The E4 allele remained rare in both groups.

*APOE* being a pivotal plasma protein involved in lipid metabolism regulation, remains central to these genetic predispositions [36]. A study that examined the correlation between *APOE* polymorphisms and lipid profiles in a Mexican Amerindian population from different ethnicities showed that exists a statistical difference in HDL-C and LDL-C levels between individuals carrying different *APOE* variants. The E2 allele had higher HDL-C levels, followed by E3 and E4 alleles [37]. Similarly, distinct associations between *APOE* gene polymorphisms and DN across different populations have been demonstrated in multiple studies [36–39]. A large meta-analysis highlighted the role of E2 allele as a risk factor for DN, in contrast to the protective roles of the E3 and E4 alleles [38]. A recent study in the Chinese Han population found that the heterozygous E3/E4 genotype and E4 allele were associated with an increased risk of T2DM [39]. Similarly, another study in the southern Chinese population identified the heterozygous E2/3 genotype and E2 allele as independent risk factors for DN, while the homozygous E3/3 genotype and E3 allele showed protective effects [40]. In line with the literature, the present study witnesses a strong association between the E2 allele and DN, as indicated by its higher frequency in DN patients compared to Controls, in adddition to the predominance of the E3 allele in the Control group. These outcomes suggest that *APOE* polymorphisms, particularly the E2 allele, is critically involved in the genetic susceptibility of DN progression.

However, from the logistic regression analysis we did not find statistically significance for difference in the risk of DN between E2 and E3 or E4 and E3 alleles. We could only show that individuals with the E2 allele had a 3.57 times higher risk of developing DN compared to those with the E3 allele and the E4 allele did not show a significant difference in DN risk. The significant correlations observed between clinical and biochemical parameters and *APOE* variants highlighted using the Pearson’s Correlation heatmap show that the E2 allele have positive correlations with elevated levels of UACR, triglycerides, LDL-C, total cholesterol, HbA1c, and plasma glucose in DN patients. In contrast, the E3 allele exhibited negative correlations with these variables in DN cohorts. Despite the established role of *APOE*, the precise impact of *APOE* polymorphisms on DN susceptibility remains nuanced and requires further elucidation. The *APOE* polymorphisms, particularly the increased prevalence of the E2 allele observed in DN cases of the present study, may contribute to the genetic susceptibility to DN in this population. While the observed biochemical and clinical associations support the role of *APOE* variants in modulating metabolic and renal parameters in DN. These insights imply on the genetic complexities underlying DN pathophysiology and emphasize the potential for personalized management strategies targeting lipid profiles and glycemic control in affected individuals with specific *APOE* genotypes. These are some of the early insights into the genetic predisposition of DN cases in context to *APOE* polymorphisms in South Indian population. Long-term pilot studies that will elucidate the precise mechanisms through which *APOE* polymorphisms influence DN susceptibility and progression are of particular interest. Further research with larger sample sizes and diverse populations are essential to validate these findings.

## Electronic supplementary material

Below is the link to the electronic supplementary material.

Supplementary Material 1

## Data Availability

The datasets generated during and/or analyzed during the present investigation are not visibly accessible due to the requirement for participant privacy but are available from the corresponding author upon judicious demand.
