# Supplementary material for: Effects of APOE isoforms in diabetic nephropathy patients of South India
Source: Acta Diabetol. 2024 Oct 17;62(4):487–97. doi: 10.1007/s00592-024-02374-2 (PMC12055913; doi:10.1007/s00592-024-02374-2)
Supplement: Supplementary file 1 — Supplementary Material 1 [file 592_2024_2374_MOESM1_ESM.docx]

**Supplementary Figures**

**
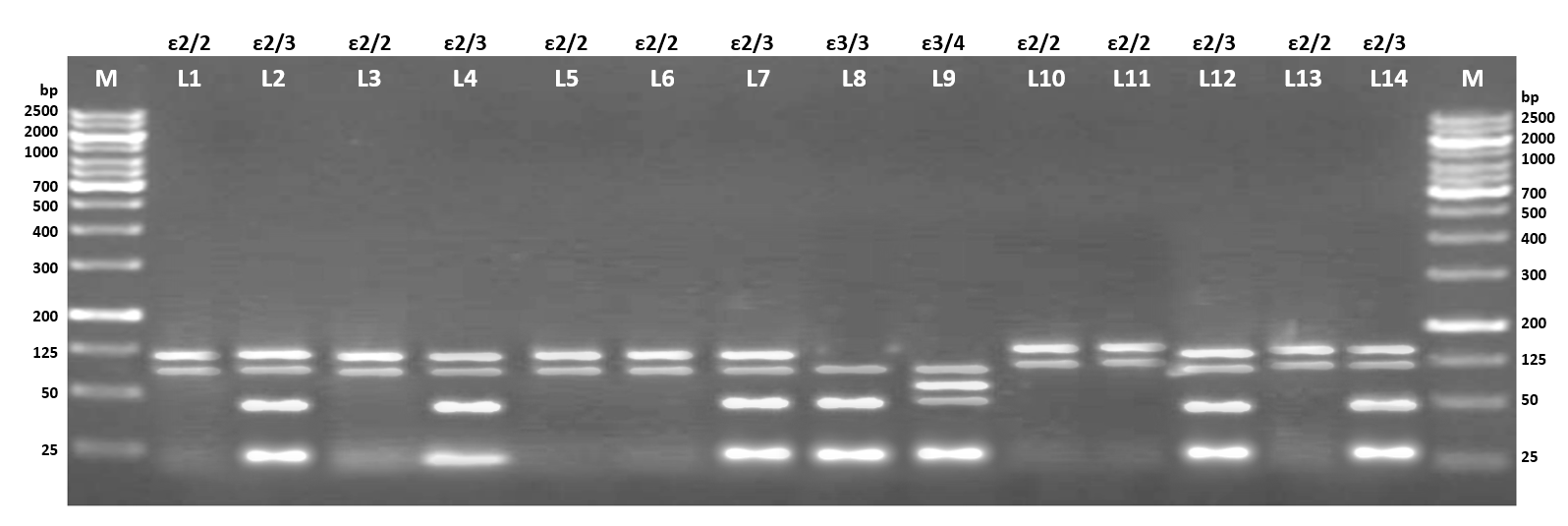
**

**Figure 1:** The picture depicts the genotype patterns observed for the 227bp *APOE* gene PCR product digested using *Hha-I* restriction enzyme, run on 3% agarose gel. The Lane 1 to Lane 14 marked as L1, L2, L3…L14 represent the series of randomly picked sample IDs confirmed for *APOE* polymorphisms. To verify the presence of specific genotype patterns of *APOE* variants: E2/2 - 104bp and 91bp; E3/3 - 91bp, 53bp and 32bp; E4/4 - 72bp, 53bp and 32bp; E2/3 - 104bp, 91bp, 53bp and 32bp; E3/4 - 91bp, 72bp, 53bp and 32bp; E2/4 - 104bp, 91bp, 72bp, 53bp and 32bp, were confirmed.
